# Physiological, anatomical and quality indexes of root tuber formation and development in chayote (*Sechium edule*)

**DOI:** 10.1186/s12870-023-04427-0

**Published:** 2023-09-07

**Authors:** Shaobo Cheng, Yuhang Liu, Lihong Su, Xuanxuan Liu, Qianwen Chu, Zhongqun He, Xiaoting Zhou, Wei Lu, Chengyao Jiang, Wangang Zheng

**Affiliations:** 1 College of Horticulture, Sichuan Agricultural University, Chengdu, 611130 China; 2 Horticulture Research Institute, Chengdu Academy of Agricultural and Forest Sciences, Chengdu, 611130 China

**Keywords:** Root tuber, Chayote, Slice structure, Physiology, Quality index

## Abstract

**Background:**

Chayote is an underutilized species of Cucurbitaceae. It is rich in nutrients such as protein, minerals, phenols and its extracts have anti-cardiovascular and anti-cancer effects, making it a versatile plant for both medicinal and culinary purposes. Although research on its root tuber is limited, they are rich in starch and have a structure similar to that of potatoes, cassava, and sweet potatoes. Therefore, they can serve as potential substitutes for potatoes and offer promising prospects as agricultural and industrial resources. However, the physiological and cellular mechanisms of chayote root tuber formation and development are still unclear.

**Results:**

In this study, we observed the growth habit of ‘Tuershao’ (high yield of root tuber). The results revealed that the tuber enlargement period of ‘Tuershao’ lasts approximately 120 days, with the early enlargement phase occurring during 0–30 days, rapid enlargement phase during 30–90 days, and maturation phase during 90–120 days. Physiological indicators demonstrated a gradual increase in starch content as the tuber developed. The activities of sucrose synthase (SUS) and invertase (VIN) showed a consistent trend, reaching the highest level in the rapid expansion period, which was the key enzyme affecting tuber expansion. Moreover, the special petal like structure formed by the secondary phloem and secondary xylem of the tuber resulted in its enlargement, facilitating the accumulation of abundant starch within the thin-walled cells of this structure. Principal component analysis further confirmed that starch content, SUS and VIN activities, as well as the concentrations of calcium (Ca), iron (Fe), and selenium (Se), were the major factors influencing tuber development. Moreover, the low temperature environment not only promoted the growth of ‘Tuershao’ tubers but also enhanced the accumulation of nutritional substances.

**Conclusions:**

These findings contribute to a deeper understanding of the formation and developmental mechanisms of ‘Tuershao’ tubers, providing valuable guidance for cultivation practices aimed at improving crop yield.

**Supplementary Information:**

The online version contains supplementary material available at 10.1186/s12870-023-04427-0.

## Introduction

Chayote (*Sechium edule*), 2n = 2x = 28, is a perennial herb climbing plant of Cucurbitaceae, which originated in Mexico and is widely planted in Japanese, Indian and southwestern China [1]. The utilization of Cucurbitaceae crops can be traced back to 15,000 years ago. Unlike other Cucurbitaceae crops, chayote has not been widely studied [2]. Chayote is rich in amino acids, proteins, phenolic compounds, flavonoid, cucurbitacin, selenium and other beneficial substances [3–7]. The whole plant of chayote can be used. Fruit is its main product. In addition, its tender tendrils are also called ‘longxucai’, which have important edible and medicinal value. However, the root tuber of chayote is often neglected. The root tuber of chayote, also known as the ichintal, is widely consumed in Latin America [8]. To date, there has been no detailed study on the formation process of the root tuber of chayote. Previous reports have suggested that the formation of root tuber may be due to higher growth vigor and excessive assimilates flowing to the root to withstand adverse growth conditions [8]. Our previous research has also confirmed that the formation of root tubers is accompanied by upregulation of starch genes and changes in sugar transporters [9]. Moreover, the nutrient-rich root tuber is being studied in depth. Previous studies have shown that the starch content in the root tuber of chayote is equivalent to that of potato, which suggested that it can be used as a substitute for starch-based crops [10, 11]. Study also found that root tuber constitutes a valuable additional source of quality fiber and carbohydrates [12]. Different sizes of root tubers have varying levels of carbohydrates and starch, and selecting the optimal period for root tubers to contain these components remains to be explored by breeders [8]. Several recent studies have shown that the starch and pectin components in the root tubers of chayote can be used to produce biologically active films in industry and have the potential to develop new food materials suitable for different processing conditions and application fields [13, 14]. In recent years, with the release of the chayote genome, transcriptome and metabolome, it will be beneficial to accelerate the research of chayote fruit and root tuber [9, 15].

Root is an important organ for plants to absorb water and nutrients, and also a key reservoir for accumulation and storage of starch, sugar and other substances. Root development and expansion is a complex physiological and biochemical process, which is regulated and affected by many factors. Genotype directly determines root characteristics, while external factors also play a key role. Environment is the external factor that directly affects root tuber expansion. The optimum temperature for the formation and expansion of sweet potato is 22–30℃, when the cambium activity of root tuber is vigorous, which is beneficial to the growth of root tuber [16]. The optimum tuberization temperature of potato is 20℃, and higher than the optimum temperature will greatly reduce tuberization [17]. When the temperature decreased from 34°/31 °C to 22°/19 °C (day/night), the storage roots of cassava increased significantly [18]. Temperature also affects the expansion of root tubers of *Curcuma longa* [19], *Puraria thomsonii* [20], *Colocasia esculenta* [21], and *Schoenoselectus nipponicus* [22]. In addition, light and water also affect the development of root tubers [23]. The root tuber will be accompanied by the change of assimilation products in the process of expansion. The change of starch, sugar, protein and cellulose content is an important evaluation index of root yield and quality [24]. Studies have shown that starch accumulates continuously in the development of root tuber, which leads to root tuber expansion. Gene editing is an effective means to improve root tuber yield [25]. Furthermore, enzyme activity plays a key role in the synthesis and metabolism of starch, sugar and other substances and in coping with environmental stress [26]. It has been reported that the activities of cell wall invertase and sucrose synthase are related to the accumulation of starch in root tuber of many plant varieties [27]. The crosstalk among phytohormones affects the development of root tuber. In recent years, many studies have shown that hormones such as auxin (AUX), cytokinin (CK), gibberellin (GA), abscisic acid (ABA) and brassinosteroid (BR) will affect the development and expansion of tuberous roots [28–30].

The cytological study can further understand the occurrence of root tuber structure. The formation of root tuber is usually regulated by the interaction between primary cambium and secondary cambium. The cells in the secondary cambium in the secondary structure of root system divide and expand continuously, which leads to the increase of root thickness and eventually forms root tuber [31]. The section results showed that the root tuber of potato was derived from stems, while cassava and sweet potato were derived from fiber roots. In addition, the root tuber of sweet potato also formed a circular secondary cambium [27]. Changes in transcription factors and genes also control the development of root tubers at the molecular level. Studies have shown that overexpression of *IbMADS1* gene will cause the adventitious roots of transgenic potato to thicken [32]. However, *IbEXP1* negatively regulates the development and expansion of sweet potato tubers. Overexpression of *IbEXP1* gene will inhibit the proliferation of xylem and cambium cells, thus inhibiting the thickening of tuberous roots [33]. Overexpression of *StSP6A* gene will inhibit the growth of potato buds, but accelerate their tuberization [27]. MADS-box, Homeobox, PEBP and other transcription factors also play an indispensable role in the information exchange and expansion of root tubers [34–36].

In this study, we studied the phenotype, physiology and biochemistry, trace elements and section observation of the different development of the root tuber of ‘Tuershao’. ‘Tuershao’ is a kind of chayote variety with high tuber yield, which has been selected by our research team for many years [9]. Its toot tuber size is significantly higher than that of ordinary chayote. Interestingly, its aboveground fruit cannot expand normally. Therefore, this study will help to broaden the basic knowledge of chayote root tuber formation and provide appropriate guidance for further harvesting of root tuber for processing and extraction.

## Results

### Root tuber growth characteristics of ‘Tuershao’

**Fig. 1:**
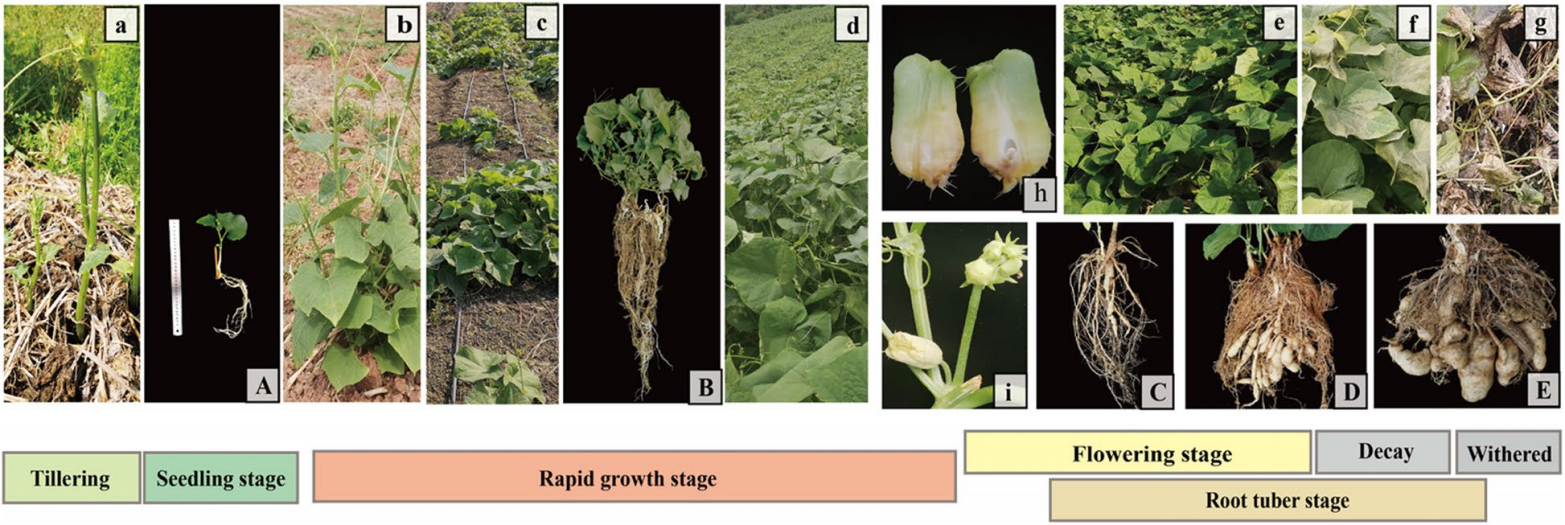
Root tuber of ‘Tuershao’ growth cycle. a: Perennial plant sprouting; b-d: Plant rapid growth stage; e: Plant flowering stage; f-g: Plant decay stage; A: Survival tiller seedling root system; B: Rapid growth period plant root system. C: Early stage of root tuber development; D: Middle stage of root tuber development; E: Maturation stage of root tuber development; h: Abortive fruit; i: Floral organ

‘Tuershao’ is a high tuber-yield chayote cultivar, which whole growth period is about 240d (Fig. 1a-g). Axillary buds of ‘Tuershao’ began to germinate and grow in the early March (Fig. 1a,). At this time, tillering seedlings were separated from perennial plants. Primary roots began to occur from callus at the base of the stem after about 20 days of culture (Fig. 1A). Withered in the upper part of the following January (Fig. 1g). The vigorous growth period of the aboveground parts was from the 30th to the 150th day (Fig. 1b-d), while the lateral roots also formed and grew rapidly (Fig. 1B). Starting to decline after 210 days (Fig. 1f). The root tuber appeared around the 160th day (Fig. 1C), rapidly expand from 180 to 220d (Fig. 1D), and mature after the decay of the aboveground parts (Fig. 1E). Flowering stage was from September to November (Fig. 1i). It is worth noting that the fruit of this plant cannot expand normally (Fig. 1h).

### Morphological and anatomical analysis of ‘Tuershao’ root system

**Fig. 2:**
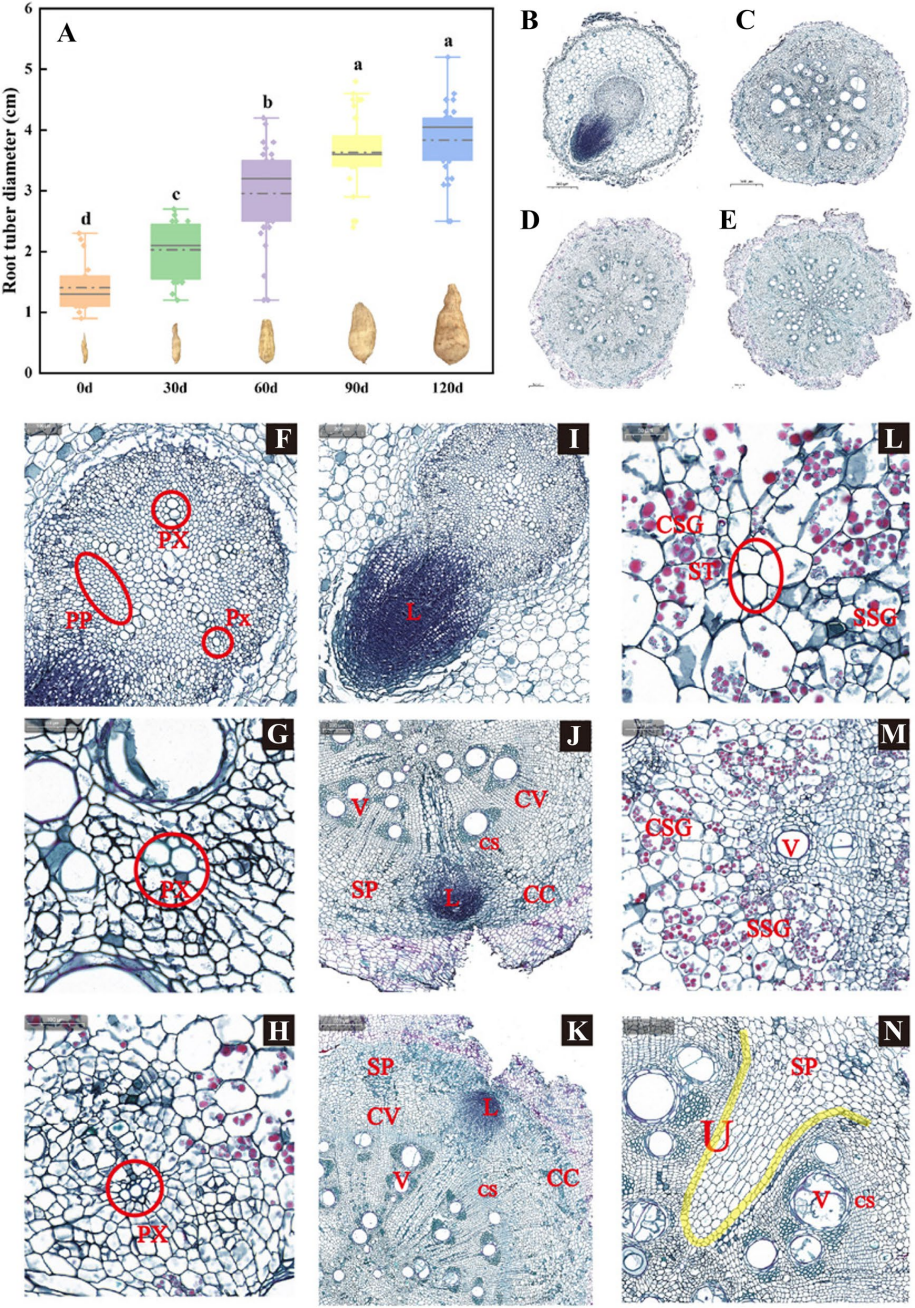
Morphological and anatomical analysis of ‘Tuershao’ root system. **A** Root tuber diameter change; Paraffin section diagram of primary root **B** fibrous root **C** root tuber **D** and stem root **E** Primary xylem of primary root **F** fibrous root **G** tuber root **H** Lateral root primordia of primary root **I** fibrous root **J** stem root **K** **L** Starch accumulation of phloem parenchyma cells; **M** Starch accumulation of xylem parenchyma cells; **N** Specificity of secondary structure. CS: Casparian strips; PP: Primary phloem; PX: Primary xylem; Px: Protoxylem; SP: Secondary phloem; CC: Cork cambium; CV: Vascular cambium; ST: Sieve tube; V: Vesse; CSG: Compound starch granule; SSG: Single starch granule; U: U-shaped vascular bundle. Different letters indicate significant differences at the level of *P* < 0.05

The root tuber began to formed at the early of October, which was taken as the starting point of root tuber expansion (0d), and the root tuber was sampled for five consecutive times at intervals of 30 days. The diameter of root tuber gradually increased with continuous growth. The difference of root tuber diameter was not significant from 90 to 120 days, and the root tuber gradually matured (Fig. 2A). The root tuber system at five different development stages mainly includes four types according to the appearance and structural differences of cross section, namely, primary young root (P1), fiber root (P2), root tuber (initial tuber root and mature tuber root, P3 and P5), stem roots (P4) (Fig. S1). The primary young root was milky white in color and only has primary structure; Fiber root, root tuber and stem root were all developed from the secondary growth of primary root, and the color was yellowish brown (Fig. S1). There were only primary young root and fibrous root in the ‘Tuershao’ root tuber of 0d and 30d, and the proportion of primary young roots was 87% and 32% respectively. Stem root and mature root tuber were found only in 90 and 120 days (Table S1).

To further understand the root tuber structure of four types of ‘Tuershao’, we made paraffin section observation (Fig. 2B-E). The cross-sectional structure of the primary root was similar to that of ordinary dicotyledonous plants, which consisted of epidermis, cortex and vascular column (Fig. 2B). The epidermis consisted of the outermost 2–3 layers of irregular cell arrangement; 8–10 layers inward were ellipsoidal parenchyma cells that constitute the cortex. The 1–2 layers close to the stele sheath were surrounded by spindle-shaped cells, which were called endodermis (Fig. 2B). The stele contained the primary xylem formed by four or five bundles of xylem radiation, with pith in the center. The primary phloem was partly distributed between the primary xylem, and its cells were small, the cytoplasm was thick, and the number was large (Fig. 2F). These tissues formed the primary structure of root tuber.

Fiber root, root tuber and stem root were derived from the secondary growth on the basis of primary roots. They were the main components of secondary structures. The secondary structure of fiber root was periderm, secondary phloem, vascular cambium, secondary xylem and primary xylem from outside to inside. The periderm consisted of three layers of cork, three to four layers of cork cambium and cork inner layer (Fig. 2C). 15 to 18 layers of larger cells formed secondary phloem, which derived inwards along the ray direction of primary xylem and nested with the secondary xylem, presented petal texture, and sieve tube and companion cell were visible. The vascular cambium was composed of 8 to 10 layers of small and tightly arranged cells. Both secondary xylem and primary xylem were 4 bundles. The Fiber root was tetrarch, and endodermis with casparian strips was obvious (Fig. 2C and G). The secondary structure of the stem root was similar to that of the fiber root. Its cork layer and secondary xylem were very developed, occupying most of the cross section of the root, and it was a pentarch root (Fig. 2E and H). The secondary phloem of root tuber was well developed, and a large number of starch bodies could be observed in secondary phloem and secondary xylem (Fig. 2D, L and M). In the secondary root system structure of ‘Tuershao’, we found U-shaped vascular bundles (Fig. 2N).

Lateral root was also an important part of the root structure of ‘Tuershao’. Slice results show that there were two main origins of lateral root. One originated from the stele sheath cell opposite to protoxylem (Fig. 2I), and the other originated from the stele sheath cell opposite to the primary phloem (Fig. 2J and K).

### Starch accumulation and distribution

**Fig. 3:**
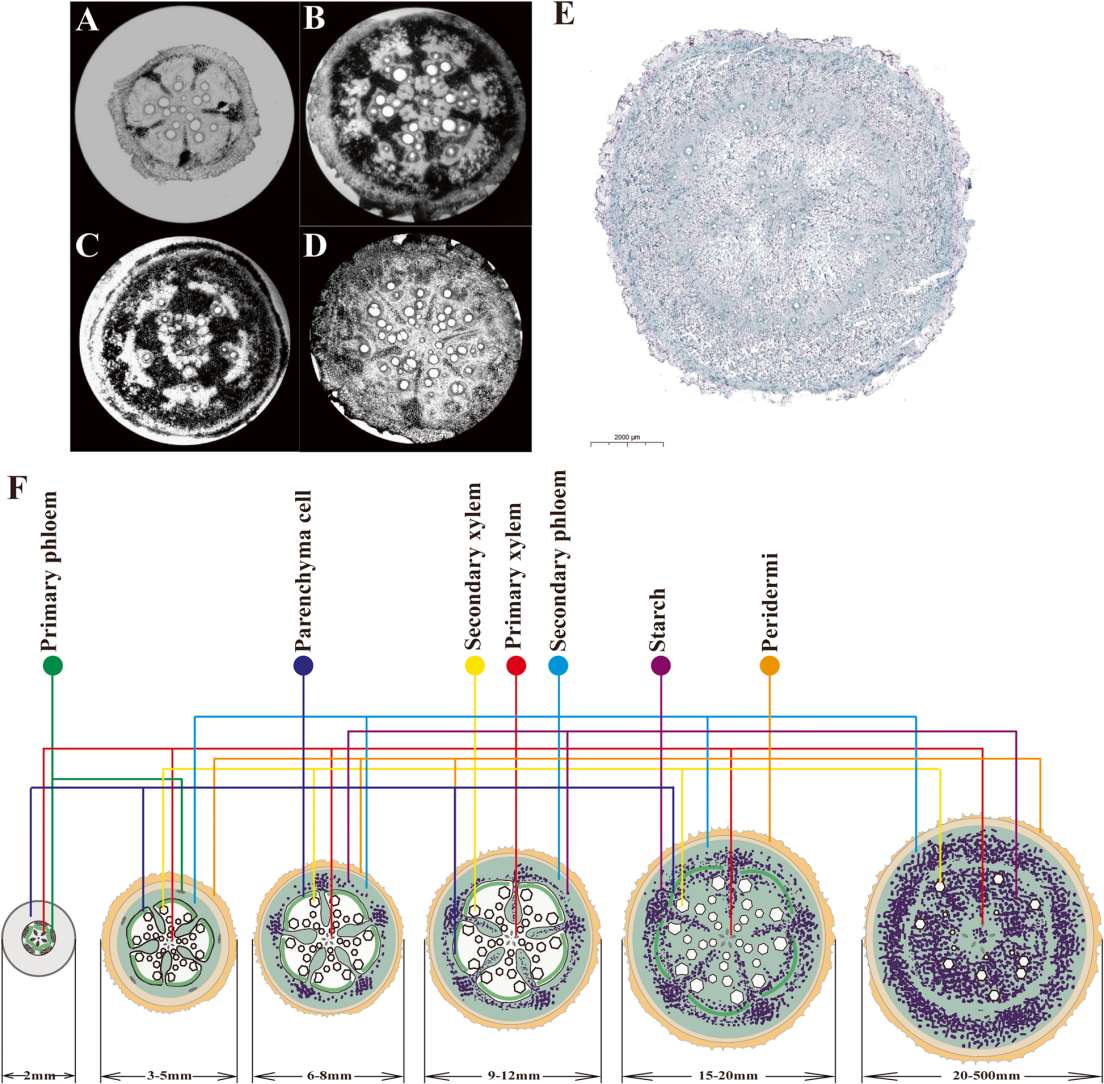
Distribution and accumulation dynamics of ‘Tuershao’ starch. Starch distribution in early root tuber **A**-**C** and stem root **D** **E** Anatomical diagram of starch accumulation in root tuber; **F** Simulated dynamic accumulation pattern of root tuber starch

We used I_2_-KI staining to observe the distribution of starch in tubers. The results showed that the stained starch granules were found in the parenchyma cells of the secondary phloem of the root tuber and stem (Fig. 3A-E). In the early root tuber, the starch granules first appeared in the secondary phloem near the sieve tube. With the development of root tubers, starch particles gradually spread outward to form a large circle (Fig. 3A-C). Subsequently, phloem cells spread to both sides, and starch grains also spread laterally. The secondary xylem was crushed, and finally a small circle was formed in the xylem. The large circle and small circle were separated by the casparian strips of the endothelium (Fig. 3E). The starch accumulation of stem root was similar to that of root tuber. The starch accumulated along the narrow phloem and finally formed a radial starch accumulation structure (Fig. 3D). We simulated the dynamic accumulation pattern of starch in root tubers by observing a large number of sections at different development stages (Fig. 3F).

### Changes of carbohydrate and related enzyme activities

**Fig. 4:**
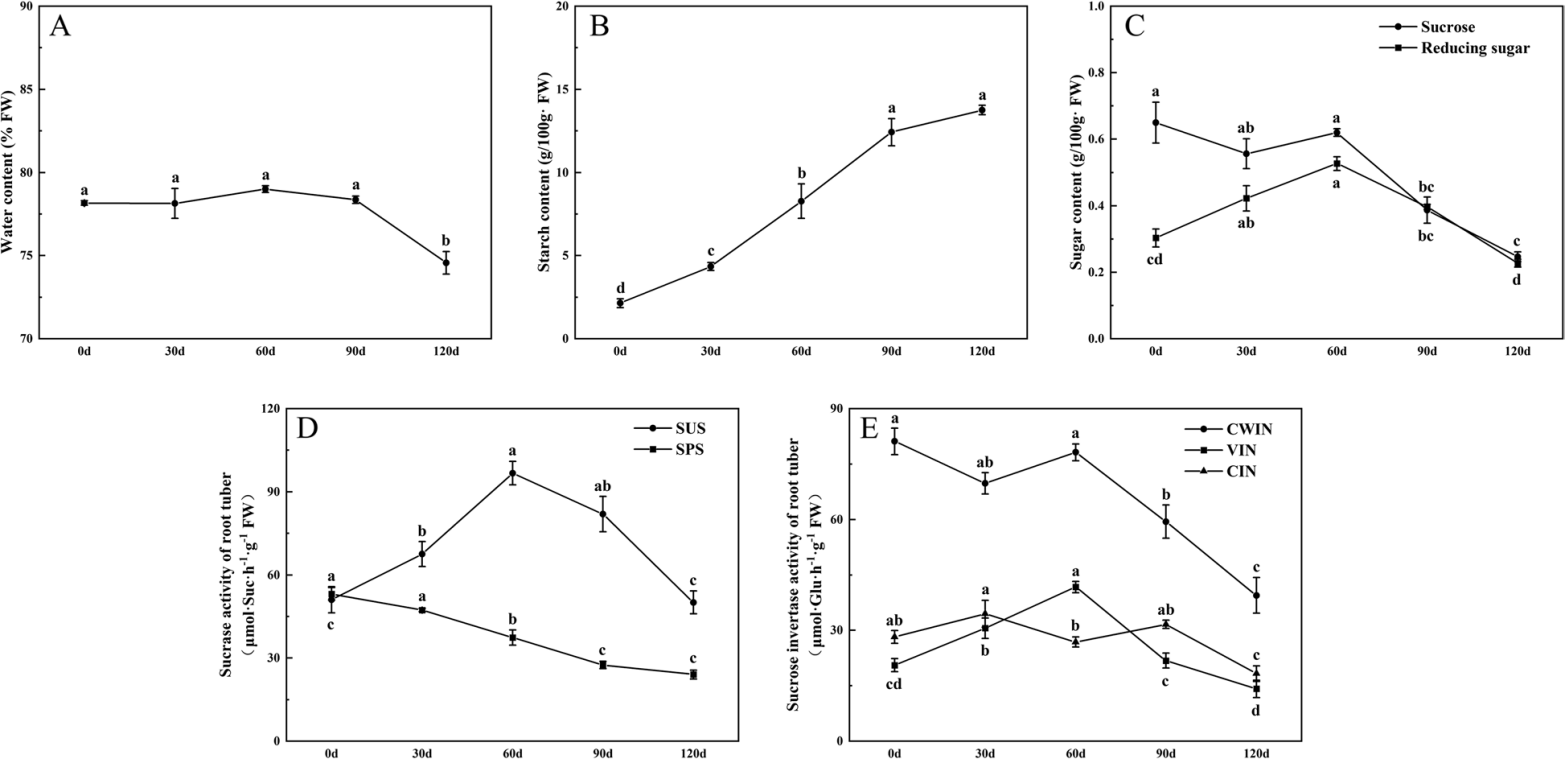
Changes of water content, carbohydrate and related enzyme activities in root tuber. **A** Water content; **B** Starch content; **C** Sugar content; **D** Sucrase activity; **E** Sucrose invertase activity. Different letters indicate significant differences at the level of *P* < 0.05

The water content of ‘Tuershao’ does not change significantly within 0–90 days, while decreases significantly on the 120 day (Fig. 4A). The starch content of ‘Tuershao’ gradually increased from 0 to 120 days, with the fastest growth in 30 to 90 days. The starch content of root tuber reached the maximum 13.76 g 100 g^−1^ FW at day 120 (Fig. 4B). The sucrose content gradually decreased with the development of root tuber, while the reducing sugar content first increased and then decreased (Fig. 4C). Furthermore, we measured the activity of sucrose metabolism-related enzymes. The activity of SUS increased first and then decreased, which reached the peak at day 60. The SPS activity gradually decreased with the development of root tuber (Fig. 4D). The activities of three sucrose invertases were in dynamic change during the development of root tuber, which CWIN had the highest activity. The CIN activity changed little during the whole root tuber development period. The VIN activity reached its highest value at day 60 (Fig. 4E).

### Nutrient content

**Fig. 5:**
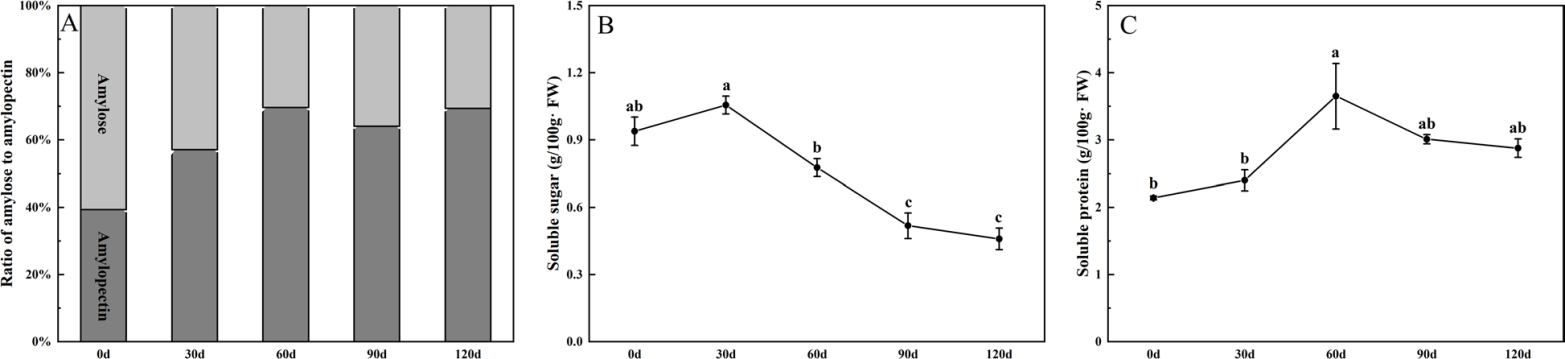
Nutrient content in root tuber. **A** The ratio of amylose to amylopectin; **B** Soluble sugar content; **C** Soluble protein content. Different letters indicate significant differences at the level of *P* < 0.05

Starch accumulation presented an S-shaped curve. We further analyzed the ratio of amylose to amylopectin. The content of amylopectin was higher than that of amylose (Fig. 5A). Soluble sugar content increased at the early stage of root tuber development and then continued to decline, reaching the highest value at day 30. (Fig. 5B). The soluble protein content of root tuber changed slowly and reached the maximum at day 60 (Fig. 5C).

### Health ingredients

**Fig. 6:**
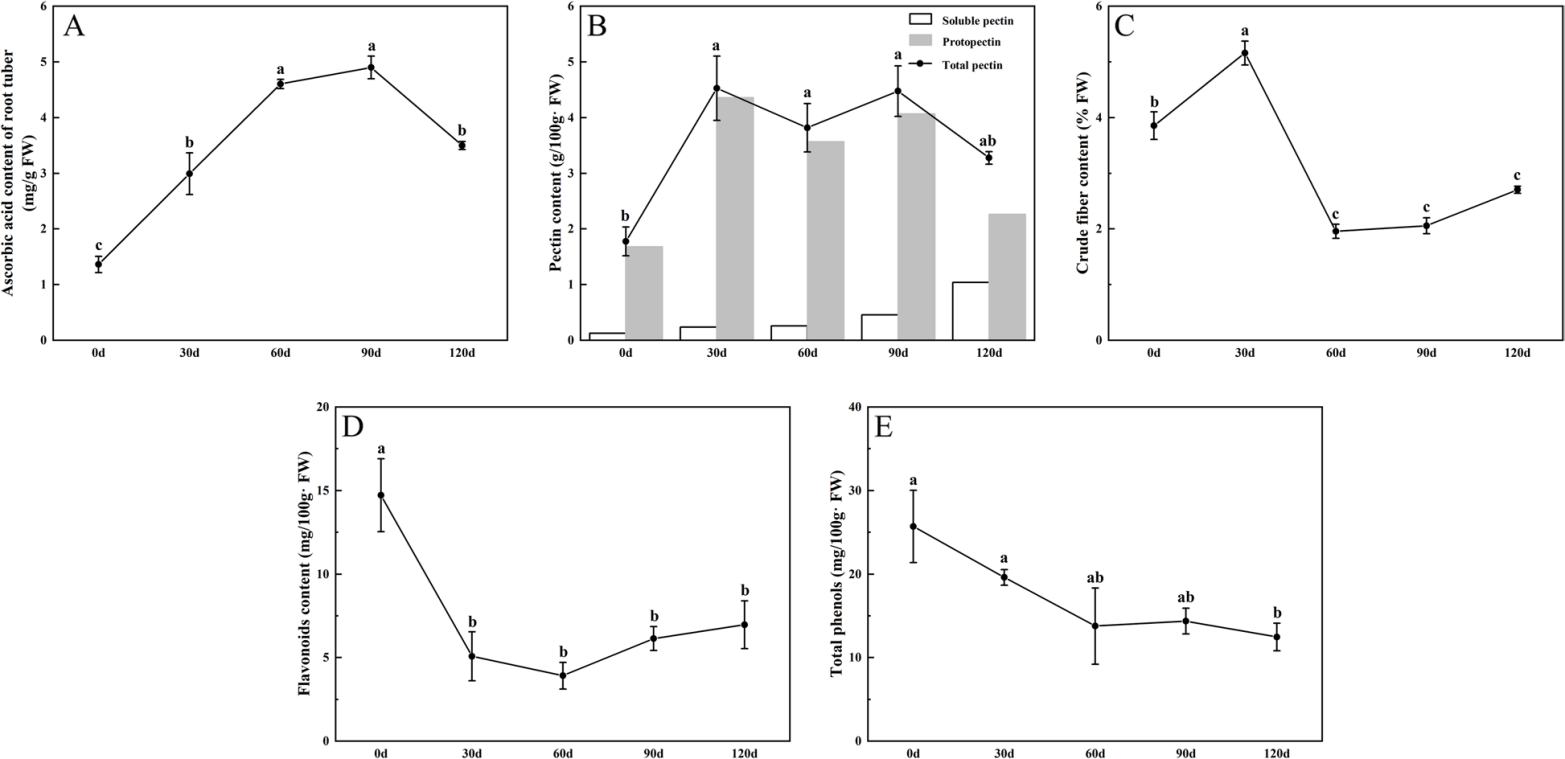
Health ingredients in root tuber. **A** Ascorbic acid content; **B** Pectin content; **C** Crude fiber content; **D** Flavonoid content; **E** Total phenol. Different letters indicate significant differences at the level of *P* < 0.05

Root tuber of ‘Tuershao’ contained ascorbic acid, pectin, crude fiber, flavonoids, phenols and other substances that were beneficial to health. Ascorbic acid content gradually increased and reached the highest level (4.89 mg g^−1^ FW) at day 90 (Fig. 6A). Soluble pectin content gradually accumulated with the development of root tuber. The total pectin content of root tuber has the same trend of change with the protopectin, and the content was the highest from the 30 to 90 day (Fig. 6B). The crude fiber content reached the highest value at day 30 and then turned to decrease (Fig. 6C). The content of flavonoids was the highest in the early stage of root tuber development. With the development of root tuber, there was no significant difference in flavonoid content (Fig. 6D). The content of total phenol decreased gradually. There was no significant change in 0–90 days (Fig. 6E).

### Content of elements

We further determined the element content in the root tuber. The content of Ca, K, Fe, Zn, Se element increased gradually with the continuous development of root tuber (Table 1). The content of all elements reaches the maximum at day120. The contents of Ca, K, Fe, Zn and Se in the mature root tuber were 3.7 times, 1.3 times, 15.3 times, 1.37 times and 2.4 times of those in the early stage of root tuber development.

**Table 1:** Changes of element content in the process of root tuber expansion

Development stages	Ca	K	Fe	Zn	Se
0d	28.9 ± 2.1	5670.8 ± 356.1	3.9 ± 0.4	5.4 ± 0.8	0.036 ± 0.001
30d	39.1 ± 0.7	5990.5 ± 298.8	27.6 ± 5.5	2.2 ± 0.0	0.044 ± 0.003
60d	51.9 ± 3.8	7034.5 ± 663.0	31.5 ± 1.0	2.0 ± 0.0	0.074 ± 0.002
90d	95.9 ± 3.7	7146.7 ± 251.1	54.6 ± 1.9	7.4 ± 0.4	0.067 ± 0.006
120d	108.4 ± 7.7	7471.7 ± 116.1	59.8 ± 2.5	7.4 ± 0.9	0.088 ± 0.004

### Multivariate statistical analysis

**Fig. 7:**
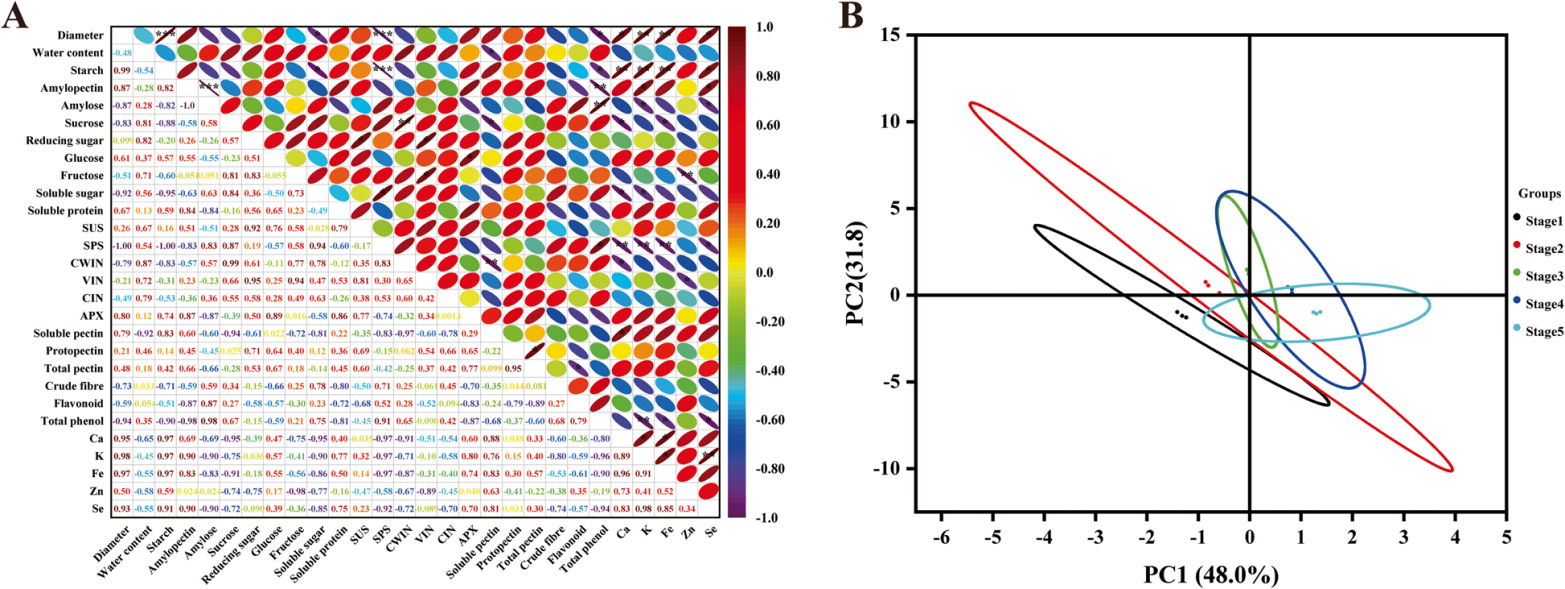
Multivariate statistical analysis. **A** Correlation analysis of all indicators. **B** PCA analysis of root tuber at different development stages

In order to better understand the relationship between changes of morphological index, carbohydrate, enzyme activities, nutrient content, health ingredients and elements content, pearson correlation analysis was used to analyze the data (Fig. 7A). The results showed that the content of starch, Ca, Fe and Se, the activity of SUS were positively correlated with root tuber diameter (*r* = 0.99***, *r* = 0.95*, *r* = 0.97**, *r* = 0.93*, *p* ≤ 0.01), while the content soluble sugar and total phenol, the activity of SPS were negative correlated with root tuber diameter respectively (*r* = -0.92*, *r* = -0.94*, *r* = -1.00***, *p* ≤ 0.01). Similarly, the starch content was negatively correlated with the content soluble sugar and total phenol, the activity of SPS (*r* = -0.95*, *r* = -0.90*, *r* =-1.00***, *p* ≤ 0.01). Sucrose content was positively correlated with CWIN activity (*r* = 0.99**, *p* ≤ 0.01). However, the contents of soluble pectin, Ca and Fe were negatively correlated with the sucrose content in root tuber (*r* = -0.94*, *r* = -0.95*, *r* = -0.91*, *p* ≤ 0.01), respectively.

PCA analysis was conducted to detect the change pattern of root tubers of ‘Tuershao’ at different development stages (Fig. 7B). PC1 and PC2 explain 48.0% and 31.8% of the variance respectively. Diameter, content of starch, Ca, Fe and Se were main key factors with positive scores in PC1. In contrast, the sucrose, total phenol content and SPS, CWIN activity were main key factors with negative scores. Reducing sugar and protopectin content, SUS and VIN activity were main key factors with positive scores in PC2, whereas flavonoid, amylose and Zn content were major factors with negative scores (Table S2). The root tuber was completely separated at different development stages.

## Discussion

Mining neglected and underutilized crop varieties was a feasible solution to cope the global food security crisis. Previous studies mainly focused on the fruit research of chayote, ignoring the root tuber. ‘Tuershao’ is different from ordinary chayote, and its ovary can't expand normally to produce fruit. It may be that the photosynthetic products above the ground are transported to the root tuber below the ground, or the root tuber absorbs too much energy. Further research is needed to prove it. The growth characteristics of ‘Tuershao’ showed that the root tuber began to form in September, expanded rapidly from October to November, and finally developed slowly. The temperature at the initial stage of root tuber expansion was 19–26 ℃. It is reported that the optimum temperature for the formation and expansion of sweet potato tubers is 22–30 ℃ [16]. This shows that the suitable temperature at the initial stage of root tuber formation is indispensable. However, the temperature at night when ‘Tuershao’ root tuber expand rapidly was about 10 ℃, which inhibits the respiration of the root tuber and thus accumulates more starch. However, the development of root tuber crops such as sweet potato and cassava is delayed at 14–22 ℃, which shows that low temperature can promote the root tuber expansion of ‘Tuershao’. It is suggested that ‘Tuershao’ is tolerant to low temperature, while normal chayote has begun to wither in November.

Root slices can help us better understand the structural changes of root tuber during expansion. Slice structure shows that potato tubers begin with stolons, and the central pith tissue expands to form arc-shaped vascular bundles. Then xylem and phloem cells are scattered throughout the whole perimedullary region to further expand the tubers, and mature parenchyma cells with large volume and rich starch granules are distributed outside the whole perimedullary region. Finally, the continuous expansion of the whole perimedullary region makes the tubers reach the final size [37, 38]. The root tuber development of ‘Tuershao’ is different from that of potato, but similar to that of sweet potato and cassava. The tuberous roots of ‘Tuershao’ developed from adventitious roots with 4–6 bundles of protoxylem, which is similar to the protoxylem where sweet potato and cassava developed into root tuber with 5 or more adventitious roots [39, 40]. The parenchyma cells between the primary phloem and primary xylem of cassava and sweet potato form arc cambium segments, and then the pericycle sheath cells are connected with the original cambium segments into a ring shape [41, 42]. However, sweet potato not only grows from its vascular cambium, but also develops many secondary circular cambia [27]. The ‘Tuershao’ forms U-shaped vascular cambium (secondary petal structure). This abnormal secondary growth may be related to plant varieties. The secondary differentiation of dicotyledons initiates from the ability of parenchyma cells located between the primary xylem and primary phloem to reactivate cell division and generate arc-shaped cambium sectors. Subsequently, pericycle cells resume division to link with the original cambium segments and form a circular ring. However, ‘Tuershao’ cambium segments do not form a circular ring but rather 4–5 curved petals. The cambial activity occurs inwardly to develop secondary xylem and outwardly for secondary phloem formation. The circular segment is pushed outward by the secondary xylem, resulting in an expanded gap between the circular segments causing the secondary phloem to be embedded in the secondary xylem and be compressed to form thin-walled cells. Therefore, this particular structure increases the number of thin-walled cells and secondary phloem, eventually leading to swell of ‘Tuershao’ root tuber through increased cell volume. At the same time, starch gradually accumulates and fills the parenchyma cells and secondary phloem, and the filling of starch is accompanied by the development of root tubers. The consistent trend of increasing root diameter and starch content also indirectly confirms this conclusion. In general, the root tuber development pattern of ‘Tuershao’ is similar to that of cassava and sweet potato. The expansion of root tuber is the result of parenchyma cell division and cambium activity.

Starch is the nutritional index of root tuber, and studies have shown that starch is the decisive factor for plants to adapt to abiotic stress [43]. With the decrease of temperature, the root tuber gradually expands and accumulates more starch to resist the low temperature environment. This may be one of the reasons why ‘Tuershao’ is more resistant to low temperature than normal chayote. In this study, stem root and root tuber of ‘Tuershao’ belong to storage roots. Starch first appeared in the secondary phloem corresponding to the primary xylem, and then gradually accumulated and spread along the secondary phloem laterally and radially, eventually leading to root swelling. Amyloplasts in root tuber usually appear as single grains. The proportion of secondary phloem in stem root is relatively small, which leads to less starch accumulation, but the special petal structure in tuber root leads to a very high proportion of secondary phloem, which eventually accumulates a lot of starch.

Sucrose phosphate synthase (SPS) is a key enzyme for sucrose synthesis, which regulates the distribution of photosynthetic products to sucrose and starch [43]. This study showed that SPS activity decreased gradually with the development of root tuber, which was contrary to the change of starch content. The higher sucrose content in the early stage of root tuber confirmed the reason of high SPS activity. Studies have shown that the root tuber development of potato, cassava and sweet potato will be accompanied by the decrease of cell wall invertase (CWIN) and the increase of sucrose synthase (SUS) activity [44–46]. In this study, it was found that SUS showed high activity in the early stage of root tuber development, decreased in the late stage of root tuber expansion, and CWIN gradually decreased, which was similar to the phenotype of enzyme activity in root tuber crops. The reason for the high SUS activity in the early stage of root tuber of ‘Tuershao’ may be that sucrose synthase mainly acts as the decomposition of sucrose in root tuber. Studies have shown that sucrose synthase can decompose and synthesize sucrose [43]. Throughout the ‘Tuershao’ swelling process, CWIN mediated the unloading of sucrose from the phloem, while SUS and INV catalyzed the breakdown of sucrose to provide glucose for starch synthesis. In parallel, VIN decomposed sucrose, thereby increasing the osmotic pressure in vacuoles, promoting cell swelling, and further expanding the volume of parenchyma cells. However, enzymatic activity for these processes declined during later stages of root development, possibly due to the low-temperature environment or saturation effects resulting from starch accumulation [25].

Notably, the root tuber of ‘Tuershao’ has a high amylose content in the early stage of development, but amylose accounts for only about 30% of the total starch in the root tuber when it matures. The high amylopectin content makes ‘Tuershao’ suitable for baking and cooking. However, its amylose content is also higher than that of most sweet potatoes, and it can be used as a starch source for processing noodles, vermicelli and other foods. In this study, the content of ascorbic acid, pectin and crude fiber in root tuber reached the maximum at 90 days, which indicated that this time could be the best harvest time for eating. However, the contents of phenols and flavonoids decreased when the root tuber matured, but they still had health care value. It may be that these secondary metabolites are transferred after synthesis, and further metabolized or degraded [47]. Previous studies showed that the content of root elements in sweet potato, cassava and pueraria lobata increased gradually with the development of root [20]. In this study, the contents of Ca, K, Fe, Zn and Se in the mature root were 3.7 times, 1.3 times, 15.3 times, 1.37 times and 2.4 times of those in the early stage, respectively. As components of various enzymes, Fe and Zn elements play an important role in enzyme activation, gene expression, plant hormone activity, carbohydrate metabolism and sugar-to-starch conversion [48]. High Fe and Zn contents in root tuber may contribute to related physiological activities. However, the high Se content in tuberous roots suggests that ‘Tuershao’ may have the function of preventing cardiovascular diseases and cancer. The nutritional quality and mineral element content in the root tuber of ‘Tuershao’ may be attributed to the failure of its aboveground fruit to develop normally. Previous studies showed that the fruit of chayote was rich in high Zn and Se elements and many beneficial substances, which cannot be stored in the aboveground fruit, and thus transported to the underground root tuber [1]. This hypothesis merits further exploration in future investigations. The nutritional value and mineral element attributes of ‘Tuershao’ root tubers may significantly increase their utilization efficiency in agriculture and industry. In fact, ‘Tuershao’ was being locally cultivated and utilized in Ya'an, Sichuan. Furthermore, flour and biscuits processed from ‘Tuershao’ had gained widespread popularity, and its extracts had displayed considerable potential as degradable films suitable for various applications [13].

Multivariate statistical analysis is helpful to better understand the results. PCA analysis can reduce multiple indicators to several indicators, and can directly observe complex indicators [49]. In this study, PCA clearly shows the specific patterns of the root tubers at different development stages. PC1 consisting of diameter, content of starch show that there were significant differences about different development stages. Correlation analysis shows that the root diameter is closely related to starch content. At the same time, the diameter is also positively correlated with the contents of Ca, Fe and Se, and negatively correlated with the nutritional indexes such as soluble sugar and total phenol, which suggests that the nutritional value can be determined according to the diameter when harvesting the root tuber of ‘Tuershao’.

## Conclusion

**Fig. 8:**
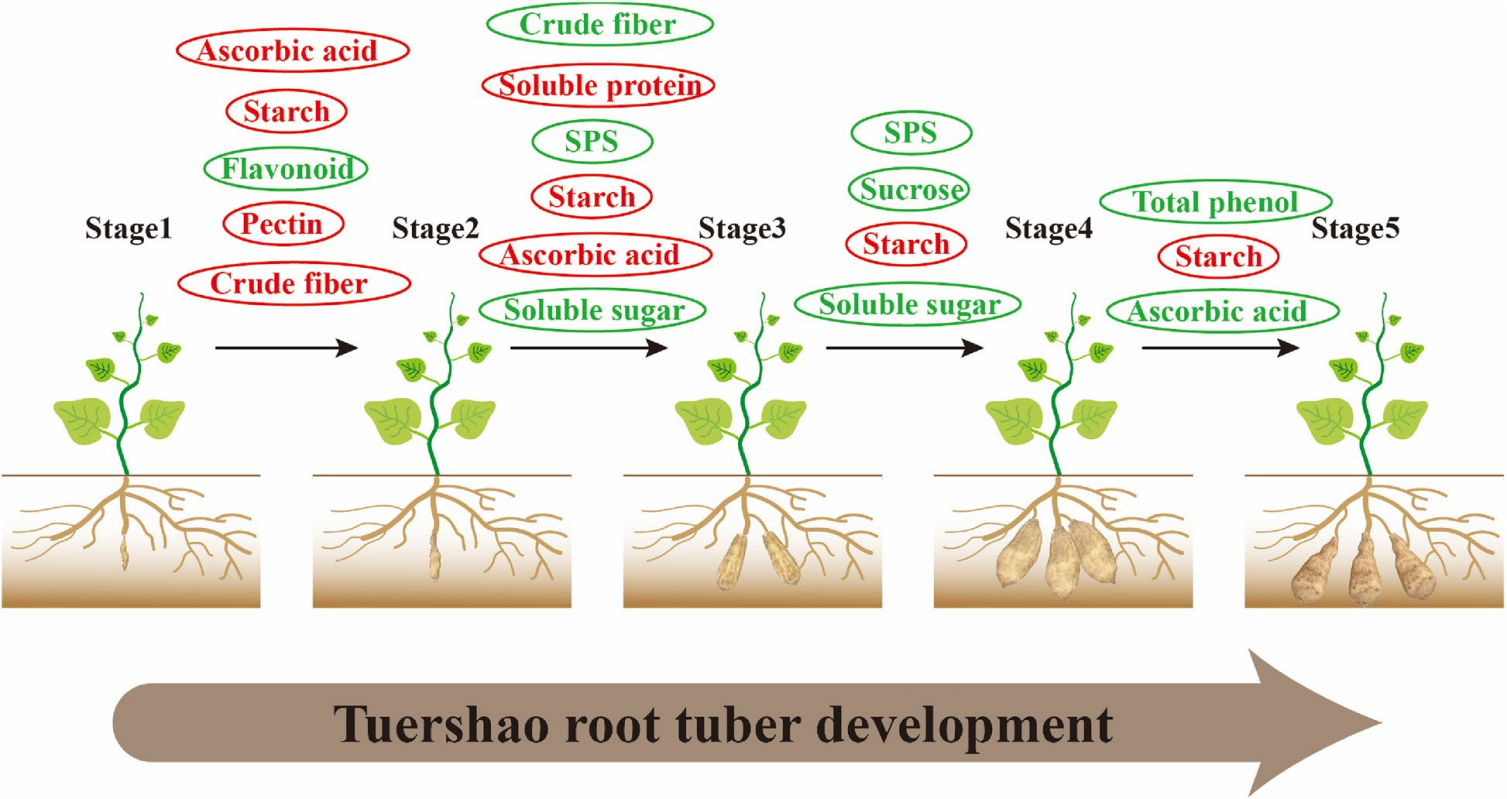
Pattern diagram of main indicators of root development of ‘Tuershao’. Red stands for rising, and green stands for falling

In this study, the mechanism of root tuber expansion of ‘Tuershao’ was explored. The secondary ‘petal’ structure produced by the proliferation and differentiation of root cambium cells and the accumulation of starch in parenchyma cells led to the expansion of tuberous roots. During the development and expansion of root tuber, many parameters change. We constructed a schematic diagram, which showed the changes of main indexes during the root tuber development of ‘Tuershao’ (Fig. 8). Our research expands the theoretical basis of root tuber formation and development of ‘Tuershao’. Moreover, it also provides an important reference point for the research of root tuber crops and the further processing and utilization of ‘Tuershao’ root tuber in the future.

## Materials and methods

### *S. edule* cultivation

‘Tuershao’ (Chuanya No.1) was a cultivar of *S. edule* which was bred to produce edible starch. However, ‘Tuershao’ unlike typical chayote plants, lacked the ability to develop fruits in the above-ground portion and instead forms enlarged underground tubers. Therefore, it served as an ideal material for investigating the mechanisms underlying tuber expand in chayote plants. ‘Tuershao’ was cultivated in Tianquan county (30°06′ N, 102°75′ E), Ya’an, Sichuan, China. ‘Tuershao’ was suitable for planting in most flat hills and high mountain areas below 1200 m above sea level in Sichuan. Tillering of perennial seedling method was employed in propagation of ‘Tuershao’. The tiller seedlings with similar growth status were transplanted into the nutrient bowl at the beginning of April. After 20 days of seedling growth, one part was transplanted into a larger planting bag (30 × 35–25 L) for continuous cultivation to determine physiological indexes, and the other part was transplanted into the field for growth observation (3 m plant spacing and 1.5 m row spacing). For the observation of the growth habits of sweet potato, we record the time when new leaves grow on the transplanted sweet potato as the 0th day. Afterwards, every 7 days, we take photos and record the development of the plant. The specific development stages include seedling stage, rapid growth stage, flowering stage, root development stage, decay stage, and withering stage. Also record the daily highest and lowest temperatures per month. Remarkably, we have permission to collect ‘Tuershao’ plant material used in the study. The ‘Tuershao’ plant comes from the Horticulture College of Sichuan Agricultural University in Chengdu and was selected by Professor Zhongqun He.

### Sample preparation

Root tubers of three ‘Tuershao’ plants were collected every 30 days at five different stages (tillering seedling new root formation stage, plant fibrous root system development stage, root tuber formation stage, root tuber expansion stage, root tuber mature stage) according to our previous planting experience [9]. Briefly, ‘Tuershao’ began to form root tuber in early October, which was recorded as the 0 day. Starting from day 0 until day 120, the root tubers of ‘Tuershao’ plants in the planting bag were washed and observed every 30 days. It is worth noting that we also observed ‘Tuershao’ before and after the 15 days that follow these five different time periods. If the root tubers had reached corresponding period, sampling was stopped. Otherwise, they will continue to grow and were sampled at the appropriate time. The aboveground and underground parts of ‘Tuershao’ plants were washed with ddwater and absorbed water. Finally, the root tubers at different development stages were photographed. Three times were performed for each experiment.

### Water content determination

After weighed the fresh weight (FW) of the samples at different development stages, put them in an oven for 30 min at 240 ℃ and then dried at 85 ℃ to determine the dry weight (DW). The Water content was measured according to the formula: WC = (FW-DW)/FW × 100% [50].

### Sugar content extraction and determination

The extraction of sugar solution was carried out according to the previously described method [51]. Root tuber of ‘Tuershao’ at different development stages was collected and dried in an oven and then grinded. Weighed 20 mg of sample into a test tube and added 10 ml of 80% alcohol. Then heated it in a 70 ℃ water bath for 50 min and transferred it to a 50 ml centrifuge tube. Absorbed the supernatant after centrifuging for 10 min, added 10 ml of alcohol to the remaining precipitate and centrifuged again. Finally, diluted the supernatant to 50 ml. Determined sucrose, reducing sugar and soluble sugar in the supernatant according to 3,5-dinitrosalicylic acid method and anthrone colorimetry. The starch content of the remaining precipitate was determined by acid hydrolysis method [52].

### Assay of enzymes related to sucrose metabolism activities

The enzyme solution was extracted according to the previous method. Weighed 0.1 g of sample and ground it, then added 1 ml of extraction solution (50 mM Tris–HCl, 10 mM MgCl2, 10 mM Vc, 2 mM EDTA, 0.1 mM PMSF, 2 mM DTT) and centrifuged it in a 2 ml centrifuge tube for 15 min at 10000 g speed. For Cell wall-bound invertase (CWIN) and vacuolar invertase (VIN), mixed 200 μl of enzyme solution with 600 μl of acetic acid-sodium acetate (PH = 4.8) and 200 μl of sucrose, and take a bath at 37 ℃ for 30 min, then added 1 ml of 3,5-dinitrosalicylic acid to react in a bath at 100 ℃ for 5 min, the absorbance was measured at 560 nm. For cytoplasmic invertase (CIN), mixed 200 μl of enzyme solution with 600 μl of acetic acid-sodium acetate (PH = 7.5), the following method was the same as above. For sucrose phosphate synthase (SPS), mixed 80 μl of enzyme solution with 20 μl of extraction solution (100 mM Hepes–KOH (pH 7.5), 10 mM UDPG, 10 mM F6P, 40 mM G6P, 5 mM MgCl_2_, 1 mM EDTA), reacted in boiling water bath for 3 min after 15 min in 30 ℃ water bath, and determined the content of sucrose produced by colorimetry. SPS activity was the amount of sucrose synthesis per gram of sample per hour. For sucrose synthase (SUS), SUS activity was the amount of catalytic sucrose decomposition per gram of sample per hour [45].

### Optical microscope analysis

Selected root tubers at different development stages and measured their diameters after cleaning. Cut a sample of about 1 cm and put it in FAA fixed solution for paraffin sectioning. Fix the sample using FAA fixative solution at 4℃ for 24 h and gradually dehydrate with 85%, 95%, and 100% ethanol solutions for 2 h. 100% ethanol needs to be treated twice. After being transparent with xylene, it is then immersed in wax and embedded, sliced using a slicer, and double stained with saffron and solid green. I_2_-KI staining was used to observe the starch distribution in the root tuber. Used the Microscope slide scanner PANNORAMIC DESK II DW (3DHISTECH Ltd., Hungary) to scan the slice, and the software Case Viewer (Sysmex Europe GmbH, Germany) was used to analyze the slice data [31].

### Elements determination

The specific method for determining the content of calcium, iron, zinc and selenium is as follows: 0.25 g of the sample was weighed and put it in a conical flask and mixed with 20 ml of nitric acid and perchloric acid solution (4:1 V/V), then sealed it with plastic wrap overnight and heated on an electric heating plate for digestion. Finally, the solution was diluted to 50 ml with deionized water. Calcium, zinc, iron and selenium were determined by flame atomic absorption spectrometry in accordance with GB 5009.92–2016, GB 5009.14–2017, GB 5009.90–2016 and GB 5009.93–2017 respectively [20].

### Determination of root tuber quality index

The method for determining the nutritional quality of root tubers was based on previous research. Determination of soluble sugar content by anthrone colorimetry, the absorbance was measured at 630 nm. The content of soluble protein was determined by Coomassie brilliant blue G-250 method, the absorbance was measured at 595 nm. The content of pectin in root tuber was determined by carbazole colorimetry, the absorbance was measured at 530 nm. Determination of amylose content in root tuber by iodine colorimetry, the absorbance was measured at 630 nm. Ascorbic acid was determined by 2,6-dichlorophenol indophenol titration. Determination of crude fiber content by acid–base digestion method. The total phenols and flavonoids were determined by ultraviolet absorption method, the absorbance was measured at the wavelength of 280 and 325 nm respectively [43].

### Statistical analysis

SPSS26.0 software was used for data collection and analysis. Duncan’s test was used to test mean comparisons. One-way analysis of variance (ANOVAs) conducted a significance analysis. All letters indicated significant differences at 0.05 level. The graphs were edited with Origin 2021 and R 4.0.3 software.

### Supplementary Information

**Additional file 1.**

## Data Availability

The data that support the results are included within the article and supplement materials. Other relevant materials are available from the corresponding authors on reasonable request.
