# Supplementary material for: Physiological, anatomical and quality indexes of root tuber formation and development in chayote (Sechium edule)
Source: BMC Plant Biol. 2023 Sep 7;23:413. doi: 10.1186/s12870-023-04427-0 (PMC10483781; doi:10.1186/s12870-023-04427-0)
Supplement: Supplementary file 1 — Additional file 1. [file 12870_2023_4427_MOESM1_ESM.docx]

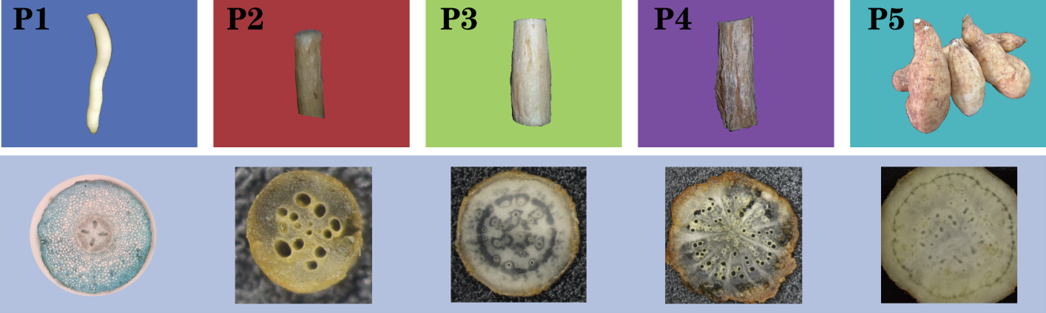


**Figure S1. Root composition and cross section of different root types of ‘Tuershao’**

**P1：primary young root；P2：fibrous root；P3：initial tuber root；P4：stem root；P5：mature tuber root**

**Table S1. Quantification of differences in anatomical structure of different types of roots**

| Root type | Primary root | Fibrous root | Stem root | Early tuber | Mature tuber |
| --- | --- | --- | --- | --- | --- |
| Number of xylem bundles | 4～6 | 4 | 4～6 | 5 ~ 6 | 5 ~ 6 |
| diameter/mm | 1.5～2.5 | 1～5 | 5～20 | 10～20 | 20～500 |
| Periderm ratio/% | 0 | 17.2 | 35 | 21.1 | 18.2 |
| Proportion of secondary xylem/% | 0 | 32.3 | 30 | 31.3 | 19.1 |
| Proportion of secondary phloem/% | 0 | 50.5 | 35 | 47.5 | 62.7 |
| Mean area of catheter/μm² | 680.8 | 25945.8 | 26860.2 | 23324.2 | 11028.8 |
| Parenchymal cell area/μm² | 225.8 | 1801.5 | 773.9 | 5164 | 58283.9 |
| Starch accumulation in phloem | - | precious few | a little | many | many |
| Starch accumulation in xylem | - | - | - | precious few | many |

**Table S2. Coefficient of variables in PCA**

| Variables | Codes | PC1 | PC2 | PC3 | PC4 | PC5 |
| --- | --- | --- | --- | --- | --- | --- |
| Diameter | X1 | 0.26878 | 0.03799 | -0.0242 | 0.01225 | -0.04885 |
| Water content | X2 | -0.15352 | 0.20206 | 0.01355 | 0.29982 | -0.21494 |
| Starch | X3 | 0.26662 | 0.01714 | -0.0142 | 0.05649 | 0.12459 |
| Amylopectin | X4 | 0.21015 | 0.19476 | -0.01673 | -0.12181 | 0.094 |
| Amylose | X5 | -0.21015 | -0.19476 | 0.01673 | 0.12181 | -0.094 |
| Sucrose | X6 | -0.23666 | 0.11303 | -0.16232 | 0.04317 | -0.1122 |
| Reducing sugar | X7 | -0.06745 | 0.31436 | -0.04765 | 0.0771 | -0.04447 |
| Glucose | X8 | 0.13185 | 0.19378 | 0.1289 | 0.41239 | -0.20541 |
| Fructose | X9 | -0.16113 | 0.24037 | -0.13642 | -0.17153 | 0.07844 |
| Soluble sugar | X10 | -0.13302 | 0.14936 | 0.17573 | -0.34488 | -0.26592 |
| Soluble protein | X11 | 0.12169 | 0.21424 | -0.34978 | -0.00842 | 0.35222 |
| SUS | X12 | 0.02798 | 0.31159 | -0.09898 | 0.21332 | 0.08446 |
| SPS | X13 | -0.26873 | -0.0163 | 0.00917 | -0.02037 | 0.17417 |
| CWIN | X14 | -0.23216 | 0.12091 | -0.15373 | 0.19823 | 0.0934 |
| VIN | X15 | -0.08846 | 0.29707 | -0.18123 | -0.06944 | 0.14583 |
| CIN | X16 | -0.14822 | 0.15741 | 0.40636 | 0.23707 | 0.18144 |
| APX | X17 | 0.18796 | 0.23185 | 0.01724 | 0.14621 | 0.01045 |
| Soluble pectin | X18 | 0.23749 | -0.12333 | -0.01985 | -0.20316 | 0.11355 |
| Protopectin | X19 | 0.03365 | 0.26259 | 0.40015 | -0.01805 | -0.0791 |
| Total pectin | X20 | 0.10234 | 0.23714 | 0.36882 | -0.03186 | 0.31027 |
| Crude fibre | X21 | -0.17896 | -0.07473 | 0.36784 | -0.29636 | 0.29981 |
| Flavonoid | X22 | -0.13375 | -0.24193 | -0.11895 | 0.2717 | 0.25268 |
| Total phenol | X23 | -0.21958 | -0.11274 | 0.05979 | 0.16249 | 0.48972 |
| Ca | X24 | 0.26458 | -0.04903 | 0.06437 | 0.07821 | 0.07496 |
| K | X25 | -0.16113 | 0.24037 | -0.13642 | -0.17153 | 0.07844 |
| Fe | X26 | 0.26215 | 0.03377 | 0.14082 | -0.01425 | 0.09028 |
| Zn | X27 | 0.15652 | -0.2157 | 0.07656 | 0.33023 | 0.07894 |
| Se | X28 | 0.24705 | 0.05112 | -0.22714 | -0.08772 | 0.15787 |
| Proportion of variations（%） |  | 47.9763 | 31.81469 | 7.32829 | 7.0029 | 2.3713 |
